# Environmental enrichment, training, and habitat characteristics of common bottlenose dolphins (*Tursiops truncatus*) and Indo-Pacific bottlenose dolphins (*Tursiops aduncus*)

**DOI:** 10.1371/journal.pone.0253688

**Published:** 2021-08-30

**Authors:** Lisa K. Lauderdale, Michael T. Walsh, Jill D. Mellen, Douglas A. Granger, Lance J. Miller

**Affiliations:** 1 Conservation Science and Animal Welfare Research, Chicago Zoological Society – Brookfield Zoo, Brookfield, Illinois, United States of America; 2 Department of Comparative, Diagnostic & Population Medicine, University of Florida, Gainesville, Florida, United States of America; 3 Biology Department, Portland State University, Portland, Oregon, United States of America; 4 Institute for Interdisciplinary Salivary Bioscience Research, University of California, Irvine, California, United States of America; Dolphin Research Center, UNITED STATES

## Abstract

In recent decades, animal welfare science has evolved to utilize a multidisciplinary approach to assess the welfare of animals in accredited zoos and aquariums. Science-based animal welfare assessments have become an essential component of management programs and widespread application is expected by animal care professionals. Management practices for bottlenose dolphins in accredited facilities incorporate several programs that potentially impact animal welfare including environmental enrichment and animal training. Additionally, habitat characteristics, such as the dimensions of the habitat, have been proposed to affect welfare. While accredited facilities are required to meet high standards of care, habitat characteristics and management practices are not standardized across locations. Knowledge and subsequent application of these practices and habitat characteristics can enhance our understanding of factors associated with positive welfare. As part of a larger study of dolphin welfare titled “Towards understanding the welfare of cetaceans in zoos and aquariums” (colloquially called the Cetacean Welfare Study), survey data were collected from 86 bottlenose dolphins in 40 habitats at 38 facilities in seven countries. The major aims of this paper are to provide general descriptive information regarding dolphin management in accredited zoos and aquariums and to provide supplemental context to the other research published from the Cetacean Welfare Study data set. This paper provides a review of current habitat characteristics and management practices at those 38 accredited zoos and aquariums. These data enabled the identification and quantification of how cetacean management practices differed between participating facilities accredited by the Alliance for Marine Mammal Parks and Aquariums and the Association of Zoos & Aquariums. Variables were selected based on their potential association with welfare including the physical habitat, environmental enrichment, and training programs. The variables were also used for subsequent research in this collection of related papers to investigate important connections between potential indicators of welfare and habitat characteristics, environmental enrichment, and training programs.

## Introduction

Animal welfare science has evolved rapidly in recent decades and this research has been used to advance evidence-based management in zoos and aquariums [1–4]. Large-scale multi-institutional studies have been conducted with a considerable number of species in order to improve husbandry, reproduction, and welfare (e.g., [5–7]). These investigations generally focus on species that are long-lived, adaptable, intelligent, and live in a variety of social structures because these attributes can raise unique management challenges. Bottlenose dolphins (*Tursiops truncatus*), with all of these characteristics, are the most common cetacean housed in zoos and aquariums [8, 9]. Accordingly, animal care professionals such as veterinarians and husbandry/training personnel at accredited facilities strive to develop and implement comprehensive management, enrichment, medical, welfare, and training programs with dolphins living in professionally managed zoo/aquarium and ocean habitats [10].

Accreditation by a professional organization such as the Alliance for Marine Mammal Parks and Aquariums (AMMPA) and the Association of Zoos & Aquariums (AZA) is an indication that an organization adopts and improves high standards of care and implements programs that specifically evaluate and manage animal welfare [11]. Accredited organizations incorporate the welfare of the individual and the group as a top priority that guides their management practices. Zoos and aquariums accredited by the AMMPA and the AZA meet or exceed standards established by experts that ensure the highest level of care is provided. The accreditation processes include the evaluation and application of welfare practices including requirements for maintaining physical and mental health, habitat, training, research, and education. While accreditation indicates facilities operate under specific standards and guidelines, dolphins under professional care experience a wide variety of physical environments and management practices.

Management practices relating to environmental enrichment and training programs are associated with dolphin behavior and welfare as a means of providing additional opportunities for mental and physical stimulation of the animals. Environmental enrichment programs are designed to enhance an animals’ habitat and encourage species-appropriate behavior through the addition of stimuli in a variety of forms [12, 13]. The goal is to promote engagement, increase behavioral diversity, provide opportunities for behavioral choice, and give the animals some control over their environment [14–17]. Program plans often include staff adding stimuli in the form of objects and problem-solving devices [18–23]. However, not all environmental enrichment is equally effective at eliciting the desired behavior [24, 25]. One method for developing a successful environmental enrichment program is a SPIDER model, which lays out a framework that includes six steps for designing successful programs: Setting Goals, Planning, Implementing, Documenting, Evaluating, and Readjusting [26]. This requires a knowledge of the current practices already implemented and recognizing the benefits of the application of science-based analysis in moving programs forward.

In conjunction with environmental enrichment, training protocols are used to facilitate optimal care. Training programs provide cognitive enrichment and physical exercise [27], evidenced by an increase in play behaviors immediately following training sessions [28]. Dolphins under professional care are also typically trained to participate in their healthcare by learning to perform medical behaviors that enable veterinary care, improving facilities’ ability to rapidly diagnose, treat, and prevent illnesses [29]. This essential practice enables staff’s ability to engage in preventative medicine, reduce the stress related to medical procedures, improve timelines for intervention, require fewer people involved in procedures, and decrease the likelihood of injuries [29, 30].

The dolphin’s habitat is another major component of environment that is related to their welfare. Habitat use is a potential indicator of the appropriateness of an environment for a species or individual of interest [31, 32]. The physical characteristics, such as length and depth, of the dolphins’ habitats have been suggested to influence behavior [33, 34]. When given free-choice to swim in areas with depths ranging from 3.96 to 8.23 m, bottlenose dolphins swam in the shallow and moderate depth areas 97.0% of the day [35]. Coastal wild dolphins also utilize the top 5 m of the water column the majority of the time [36–39]. In terms of length, the horizontal dimension of the habitat has been associated with increases in traveling time and nursing behaviors [33, 40].

The goal of this paper is to provide a review of current management practices and habitat characteristics at a large number of accredited bottlenose dolphin-holding facilities with the goals of publishing general information regarding attributes of habitats and management as well as to provide supplemental context to the other research published from the Cetacean Welfare Study data set. This research was focused on three factors that are potentially associated with welfare: characteristics of the physical habitat, environmental enrichment programs, and training programs. The direct and synthesized metrics described here were also leveraged for use in the other studies of cetacean welfare included within this collection.

## Materials and methods

### Ethics statement

This study was authorized by the management at each participating zoo and aquarium and, where applicable, was reviewed and approved by research committees. In addition, the study protocol was reviewed and approved by the U.S. Navy Marine Mammal Program Institutional Animal Care and Use Committee #123–2017.

### Subjects and facilities

The current study is one component of a larger study entitled “Towards Understanding the Welfare of Cetaceans in Zoos and Aquariums” (colloquially called the Cetacean Welfare Study). Zoos and aquariums that were accredited in 2017 by the AMMPA or the AZA were eligible for participation in this part of the Cetacean Welfare Study provided they cared for common bottlenose dolphins or Indo-Pacific bottlenose dolphins (*Tursiops aduncus*). To create a balanced representation of dolphins under professional care, two dolphins living in each of the 40 habitats were selected to participate in this portion of the study using a semi-random sampling design. Six dolphins were only able to participate in one of the two five-week periods of data collection, so a different individual was substituted for the second data collection period resulting in a total of 86 individual participants over the two data collection periods.

### Management survey

Data were derived from a survey of management practices and training logs that were completed by animal care staff from zoos and aquariums participating in the multi-institutional study of cetacean welfare. The survey questions were developed by a group of current experts in animal welfare, cetacean management, and veterinary medicine. Links to complete the online management survey were sent to participating facilities in July 2018. One survey was completed per dolphin. Surveys for the additional six dolphins added to the 2019 data collection period were completed in 2019. Respondents answered the survey questions related to the focal dolphin’s experiences over the five-week data collection period. Surveys were completed by animal care and management staff that worked directly with the focal dolphins. The management survey was embedded with the focal dolphin’s name to ensure responses were specific to each observed dolphin. Questions were presented conditionally based on the previous response. The management survey consisted of questions related to the habitat characteristics, individual dolphins demographic information and social environment, training programs, and environmental enrichment programs with respect to the focal animals. The survey questions related to the variables presented here are in S1 File. The Dolphin Presentations, Interaction Programs, and Training Duration variables were obtained from training logs that were completed by animal care staff which included the time, duration, and type of each training session. Response reports were stored upon submission on a secure server and were de-identified for analysis.

### Statistical analyses and variable creation

Descriptive statistics were calculated for responses to the questions in the demographic, environmental enrichment program, training program, and habitat characteristics sections of the survey. In addition, information from the management survey was used to create a number of variables that integrated multiple questionnaire responses into a single, representative score quantifying selected aspects of the daily lives of the bottlenose dolphins. The direct and synthesized metrics generated from these data will also be leveraged for use in other research published from this data set. Variable descriptions and the variable creation process are described below.

#### Variable descriptions

*Sex*. Sex of the dolphin.

*Age*. Age of the dolphin in years based on the known or estimated date of birth at the onset of the data collection period.

*Enrichment program index*. Respondents rated the frequency with which they engaged in several evaluative aspects of their enrichment programs. These included how often a team set enrichment goals, how frequently a team recorded when enrichment was provided, how often enrichment was evaluated, and how often the team adjusted the enrichment. Creating goals and setting goals were highly correlated so the creating goals question was dropped from the analysis. A principle components analysis using polychoric correlations was used to reduce the four variables into a single component that explained 56.84% of the variance.

*Enrichment diversity index*. Staff at each facility listed the total number of days that they provided enrichment from any of the 22 categories given on the management survey. Enrichment categories included: above water play/scuba play, balls/buoys, boomer/beach balls, bubble machines, changing conspecifics, dead fish, feeder balls/spools, foam rollers/bats/sticks, hula hoops, ice/gelatin, kayaking/Zorb balls, Legos/dive bricks, live fish, mats/sleds/ice bergs, mirror/television/movies, noodles, puzzle feeders, rub ropes/seaweed boas, tubs, underwater music/sounds, underwater window play, and water spray/brush boards. Respondents classified the enrichment in each applicable category as floating, sinking, or both for their facility. Data were converted to proportions of total number of days provided for each enrichment category and then a diversity index was created using the Shannon diversity index [41]. Diversity is notated as *H*, with higher values signifying a greater number of enrichment types and/or a more even distribution of enrichment types [42]. The Shannon index (*H*) is calculated as

$$H=-\sum_{i=1}^{s}p_{i}\;ln\;p_{i}$$

where *p*_*i*_ is the proportion of the category.

*Night time enrichment*. The mean number of nights in a week that enrichment was provided to the dolphins at the facility during the data collection period.

*Enrichment schedule*. Description of how enrichment was scheduled during the data collection period based on predictable, semi-random, or random categories.

*Frequency of new enrichment*. The frequency with which the facility provided the dolphins with new types/forms of enrichment categorized as weekly/monthly, twice a year, or yearly/year plus.

*Dolphin presentations*. The mean number of dolphin presentations that an individual dolphin participated in each week. Dolphin presentations were educational programs viewed by the public from a distance (e.g., in stands or a stadium).

*Interaction programs*. The mean number of interaction programs that an individual dolphin participated in each week. Interaction programs are formal training sessions in which small numbers of guests were in the water or dockside.

*Training duration*. The mean amount of time (in hours) each dolphin was interacting with animal care professionals during training sessions for husbandry, presentations, interaction programs, research, or other activities each week.

*Maximum number of interaction guests*. The maximum number of guests allowed to participate in a single interaction program during the data collection period.

*Training schedule*. Description of how training sessions were scheduled based on predictable or semi-predictable categories.

*Day time spatial experience*. Each facility listed the total volume of water for each gated area of their dolphin habitat. Staff provided the mean duration of time that the dolphin spent in those areas during the day (staff working hours). This variable was created by multiplying the volume of water from each area that the dolphin spent time in by the proportion of time they had access to those areas. The resulting values were then summed for all areas throughout the day.

*Night time spatial experience*. The facilities listed the total volume of water for each gated area of their dolphin habitat. Staff provided the mean number of nights that the dolphin had access to those areas. This variable was created by multiplying the volume of water from each area that the dolphin spent time in by the number of nights they had access to those areas. The resulting values were then summed for all areas at night.

*24 hour spatial experience*. Each facility listed the total volume of water for each gated area of their dolphin habitat. Staff provided the mean duration of time that the dolphin spent in those areas during the day (staff working hours) and number of nights they had access to each area. This variable was created by multiplying the volume of water from each area that the dolphin spent time in by the proportion of time/number of nights that they had access to those areas. The resulting values were then summed for all areas over twenty-four hours.

*Length*. The longest dimension of any area that the dolphin had access to during daytime hours.

*Depth*. The deepest depth of any area the dolphin had access to during daytime hours.

*Habitat type*. Habitats were characterized as either a professionally managed zoo/aquarium habitat or professionally managed ocean habitat. Professionally managed zoo/aquarium habitats were fabricated habitats with or without exposure to weather patterns. Professionally managed ocean habitats were cordoned off sections of coastal ocean, bays, lagoons, or waterways.

*Number of habitats*. Total number of areas the dolphin had access to during daytime hours.

*Social management*. Categorization of the social management practices experienced by a dolphin during the data collection period characterized as Same Group, Split/Reunited, or Rotated Subgroups. Dolphins in the Same Group category were managed in a single group with consistent members. Dolphins in the Split/Reunited category were managed in a group that was split into smaller subgroups during the day and were reunited into one group at night. Dolphins in the Rotated Subgroups category were managed as subgroups with rotating members but were never united as one group.

*Neighboring conspecifics*. Categorization indicating if the dolphin had visual and auditory access to other dolphins without possibility of physical contact during the data collection period.

Descriptions of the final independent variables created from the management survey and training logs are given in Table 1.

**Table 1:** Independent variables created from the management survey and training logs.

Variable	Definition	Values	Type of Variable
***Demographic***			
Sex	Sex of the dolphin	Male/Female	Factor
Age	Age of the dolphin	Years	Covariate
***Environmental Enrichment***			
Enrichment Diversity Index	Enrichment diversity index was created using the Shannon diversity index on the mean number of days each enrichment is provided at the facility	Index	Covariate
Enrichment Program Index	Enrichment program index is a standardized factor score created with scores on frequency of enrichment program components used at the facility using a polychoric PCA	Index	Covariate
Night Time Enrichment	Mean number of nights in a week that enrichment was provided to the dolphins at the facility	Number of Nights	Covariate
Enrichment Schedule	Categorical value indicating how enrichment was scheduled at the facility	Predictable, Semi-Random, Random	Factor
Frequency of New Enrichment	Categorical frequency that a facility provided the dolphins with new types/forms of enrichment	Weekly/Monthly, Twice a Year, Yearly/Year+	Factor
***Training***			
Dolphin Presentations	Mean number of dolphin presentations an individual dolphin participated in each week	Mean Number of Presentations	Covariate
Interaction Programs	Mean number of dolphin interaction programs an individual dolphin participated in each week	Mean Number of Interactions	Covariate
Training Duration	Mean amount of time each dolphin interacted with an animal care professional for presentations, interaction programs, training sessions, research, or other training activities each week	Hours	Covariate
Maximum Number of Interaction Guests	Maximum number of participants allowed for an interaction program for that facility	Number of Participants	Covariate
Training Schedule	Categorical variable indicating if the training schedule for the dolphins at that facility was predictable or semi-predictable	Predictable, Semi-Predictable	Factor
***Habitat Characteristics***			
Day Time Spatial Experience	Proportionate volume of water the dolphin had access to based on the percentage of daytime hours spent in different habitats in each five-week data collection period	Megaliter	Covariate
Night Time Spatial Experience	Proportionate volume of water the dolphin had access to based on the percentage of night time hours spent in different habitats in each five-week data collection period	Megaliter	Covariate
24 Hour Spatial Experience	Proportionate volume of water the dolphin had access to based on the percentage of hours throughout the entire day spent in different habitats in each five-week data collection period	Megaliter	Covariate
Length	The maximum straight length in any direction across any habitat the dolphin had access to in each five-week data collection period	m	Covariate
Depth	The maximum depth for any habitat the dolphin had access to in each five-week data collection period	m	Covariate
Habitat Type	Categorical variable indicating the dolphin was in a professionally managed zoo/aquarium habitat or a professionally managed ocean habitat	Zoo/Aquarium, Ocean	Factor
Number of Habitats	Maximum number of habitats (different enclosures) dolphin had access to in daytime hours during each five-week data collection period	Number of Habitats	Covariate
Social Management	Categorical variable indicating the type of social management practice for a dolphin during each five-week data collection period	Same Group, Split/Reunited, Rotated Subgroups	Factor
Neighboring Conspecifics	Categorical variable indicating if the dolphin had visual and auditory access to other dolphins without possibility of physical contact during each five-week data collection period	No, Yes	Factor

## Results

### Demographic variables

Responses to the survey were received from all participating facilities. In total, survey data were collected for 82 common bottlenose dolphins and 4 Indo-Pacific bottlenose dolphins living at 38 facilities (in 40 habitats). Participating habitats were located in Bermuda (n = 1), Hong Kong (n = 1), Jamaica (n = 2), Mexico (n = 18), Singapore (n = 1), Spain (n = 1), and the United States (n = 16). Descriptive statistics for the demographic variables are given in Table 2.

**Table 2:** Demographic variables.

Variable	Category	n	Mean	Median	SD	Min	Max	Inter-Quartile Range
Sex	Male	52	-	-	-	-	-	-
	Female	34	-	-	-	-	-	-
Age (years)		86	18.24	15.00	10.91	3.00	48.00	15.00

### Environmental enrichment variables

**Fig 1:**
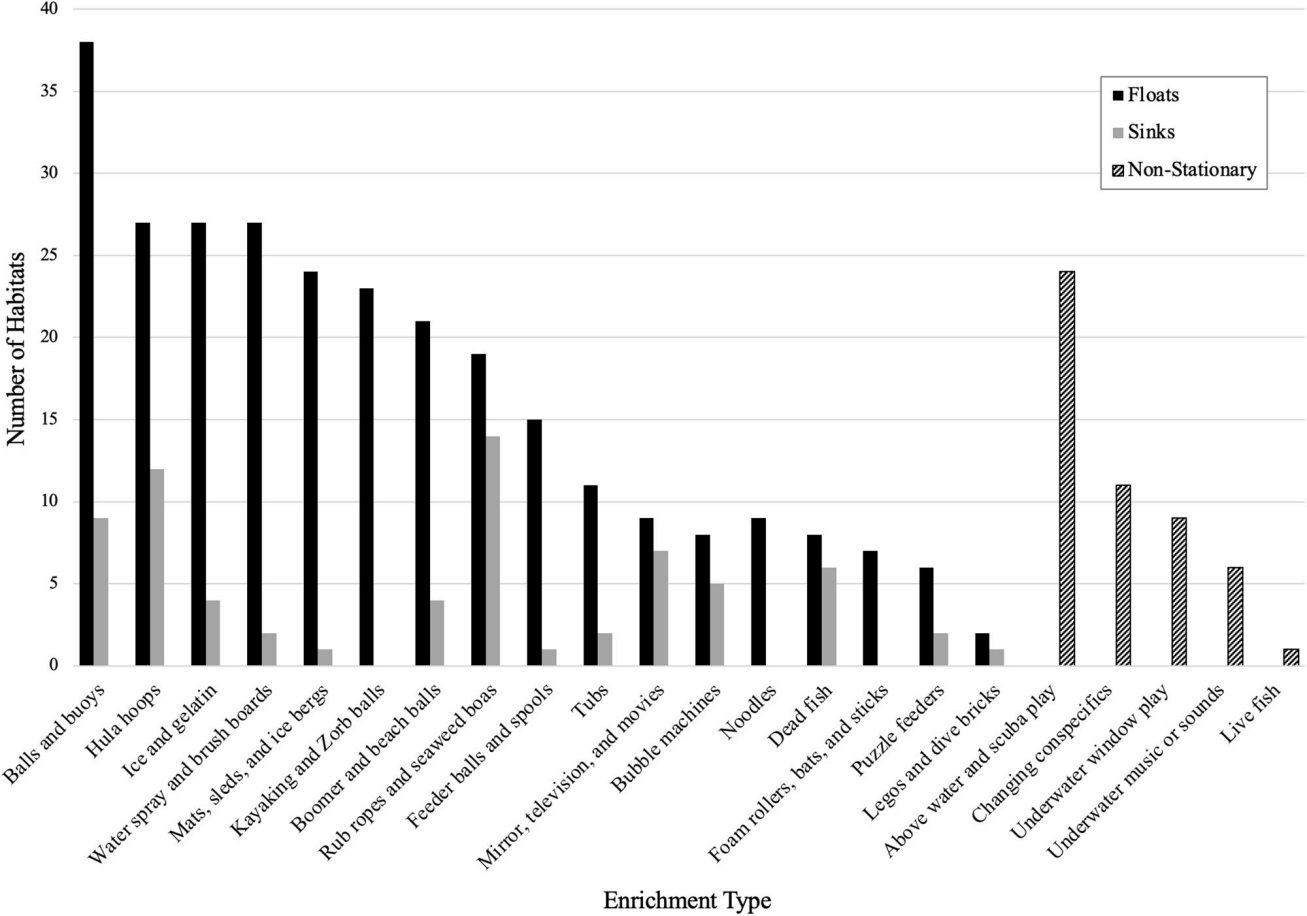
Number of habitats that were provided each type of enrichment.

Dolphins in 39 of the 40 habitats received environmental enrichment from at least one of the categories listed on the management survey. The number of habitats where each enrichment category was utilized is displayed in Fig 1. Balls and buoys were the most commonly used type of enrichment. In the majority of programs (75.6%), environmental enrichment was provided on a semi-random schedule. New enrichment was added at least once a month at 62.8% of habitats. Values for the direct and created independent variables related to environmental enrichment are given in Table 3. The enrichment diversity index was a value representing the diversity of types of enrichment used in the enrichment program, where higher values signifed a greater number of enrichment types and/or a more even distribution of enrichment types. The enrichment program index variable was a value created from survey responses rating the frequency with which the facility engaged in several evaluative aspects of their enrichment programs. See [43–46] for details on how these variables relate to indicators of welfare.

**Table 3:** Environmental enrichment variables.

Variable	Category	n	Mean	Median	SD	Min	Max	Inter-Quartile Range
Enrichment Diversity Index		86	1.79	1.91	0.64	0.00	2.59	0.80
Enrichment Program Index		86	0.05	-0.08	1.01	-1.10	2.41	1.26
Night Time Enrichment		86	2.01	0.00	2.86	0.00	7.00	4.75
Enrichment Schedule	Predictable	16	-	-	-	-	-	-
	Semi-Random	62	-	-	-	-	-	-
	Random	8	-	-	-	-	-	-
Frequency of New Enrichment	Monthly / Weekly	54	-	-	-	-	-	-
	Twice a Year	22	-	-	-	-	-	-
	Yearly / Year +	10	-	-	-	-	-	-

### Training variables

All zoos and aquariums participating in this study had a training program in place for their dolphins that included multiple daily training sessions. The dolphins all participated in training sessions not related to public engagement, interaction programs, and/or public presentations. With respect to training sessions viewed by the public (i.e., interaction programs and public presentations), fifty-six of the 86 dolphins in this study only participated in interaction programs with guests, five of the 86 dolphins only participated in public presentations, and twenty two of the 86 dolphins participated in both presentations and interactions. The remaining three of the 86 dolphins did not participate in presentations or interaction programs (i.e., none of their training sessions were related to public education). Most training programs used semi-predicable schedules (62.8%) to determine when training sessions would occur. Values for the direct and created independent variables related to training programs are given in Table 4.

**Table 4:** Training variables.

Variable	Category	n	Mean	Median	SD	Min	Max	Inter-Quartile Range
Dolphin Presentations		86	2.65	0.00	6.17	0.00	27.80	0.75
Interaction Programs		86	15.36	15.80	10.30	0.00	40.20	16.75
Training Duration (hr)		86	14.67	12.98	8.07	2.85	39.86	8.50
Maximum Number of Interaction Guests		86	12.16	10.00	9.43	1.00	30.00	10.50
Training Schedule	Predictable	32	-	-	-	-	-	-
	Semi-Predictable	54	-	-	-	-	-	-

### Habitat characteristic variables

**Fig 2:**
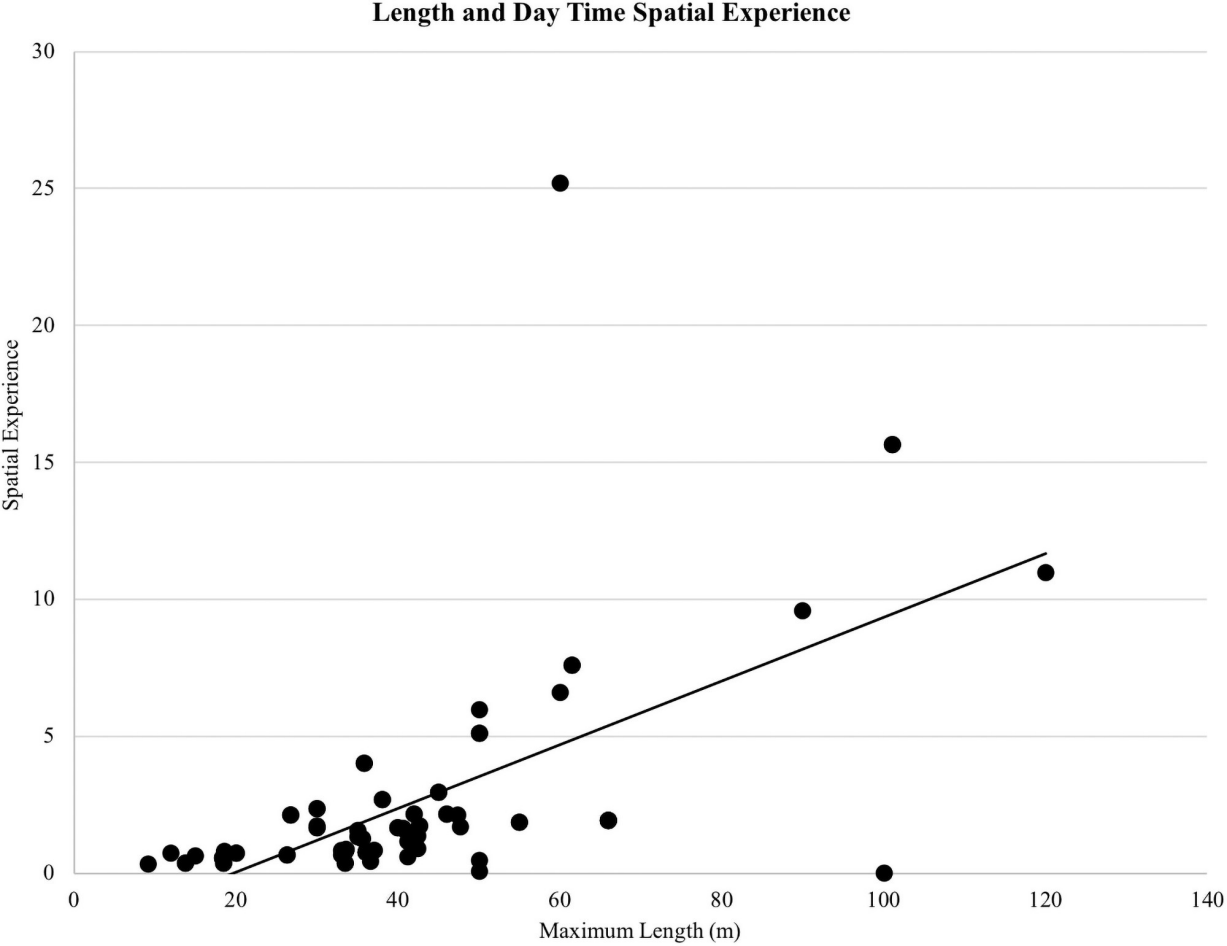
Maximum length plotted against the day time spatial experience variable. Day Time Spatial Experience was the proportionate volume of water the dolphin had access to based on the percentage of daytime hours spent in different habitats in each five-week data collection period.

**Fig 3:**
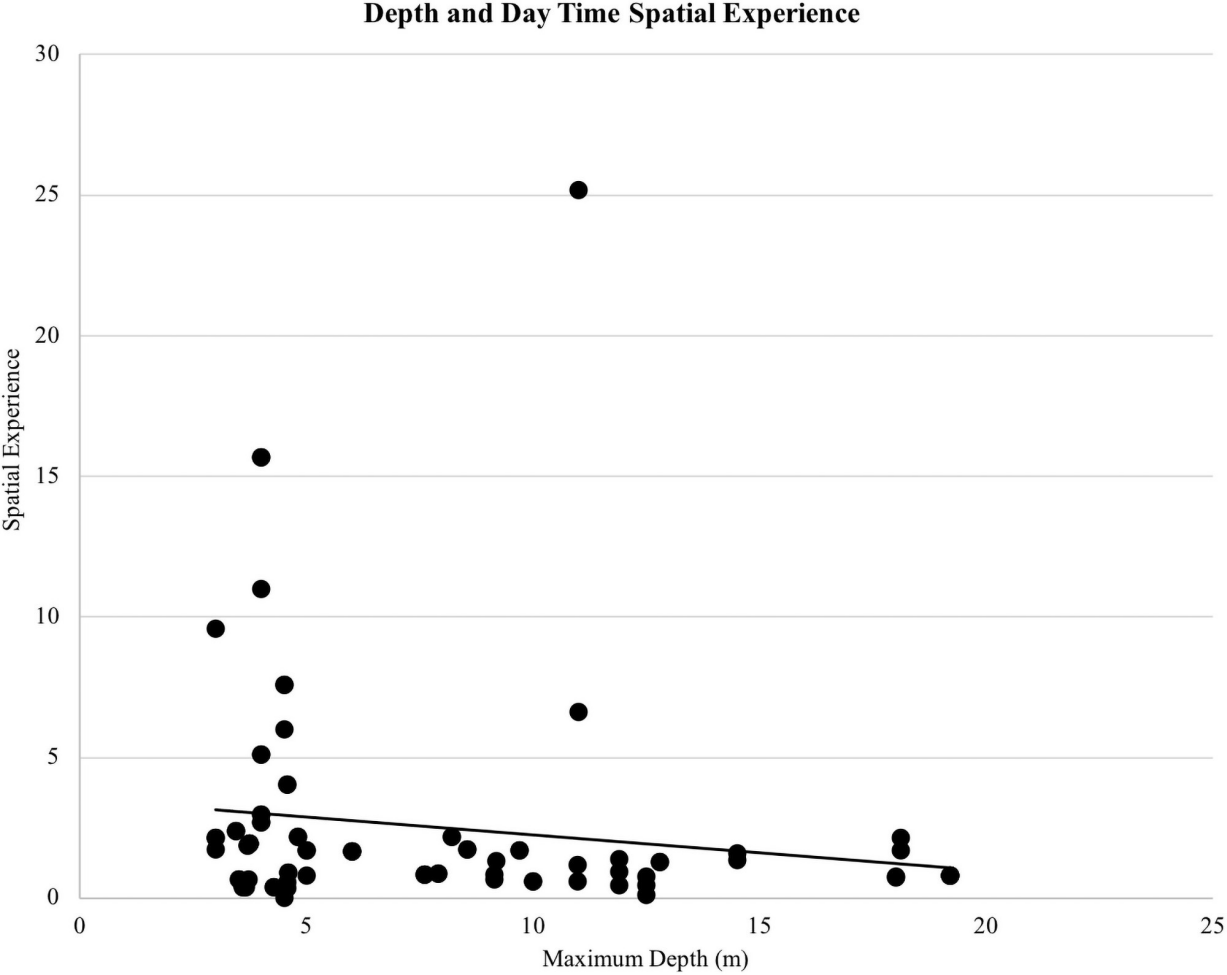
Maximum depth plotted against the day time spatial experience variable. Day Time Spatial Experience was the proportionate volume of water the dolphin had access to based on the percentage of daytime hours spent in different habitats in each five-week data collection period.

**Fig 4:**
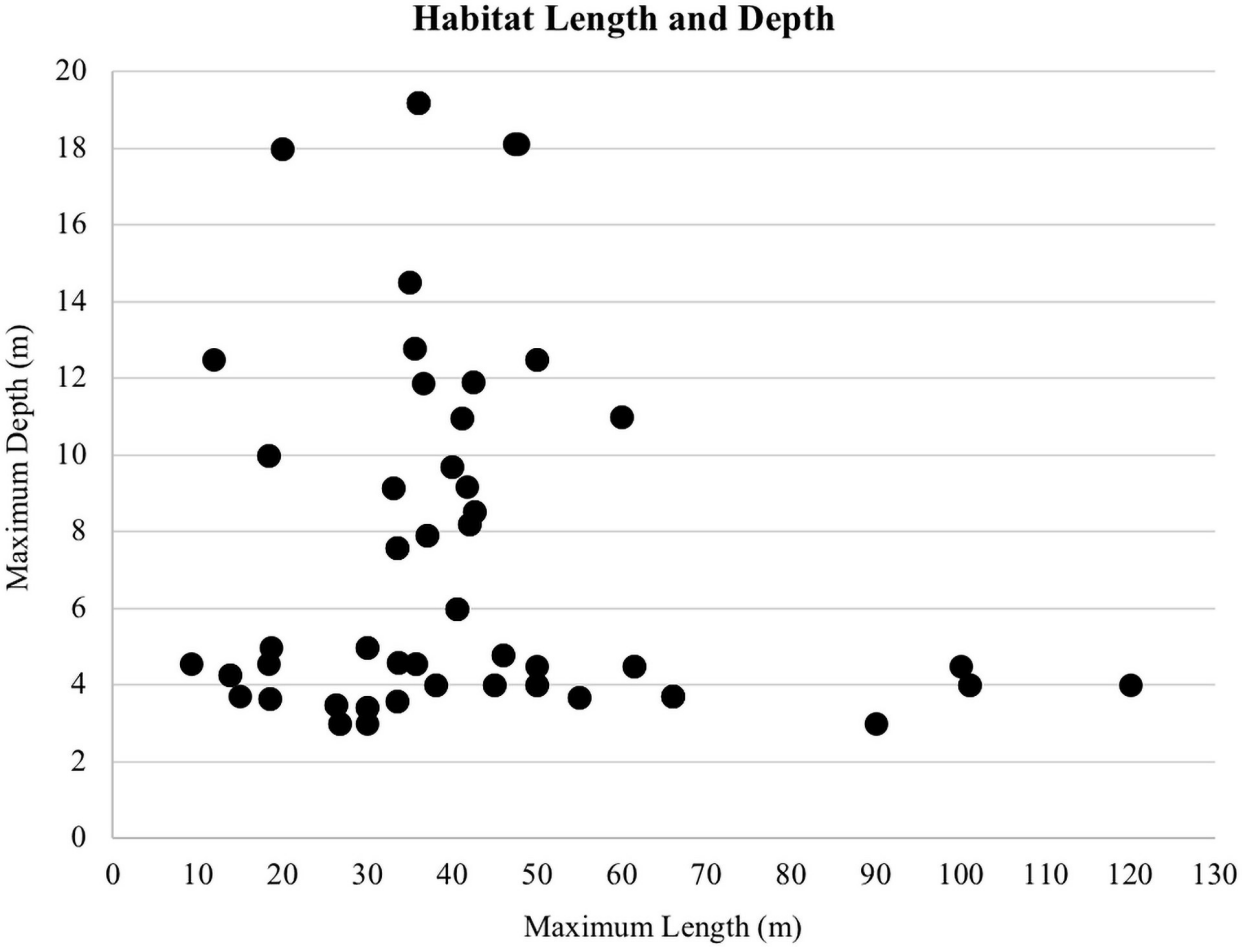
Maximum length plotted against maximum depth for the 40 habitats.

**Fig 5:**
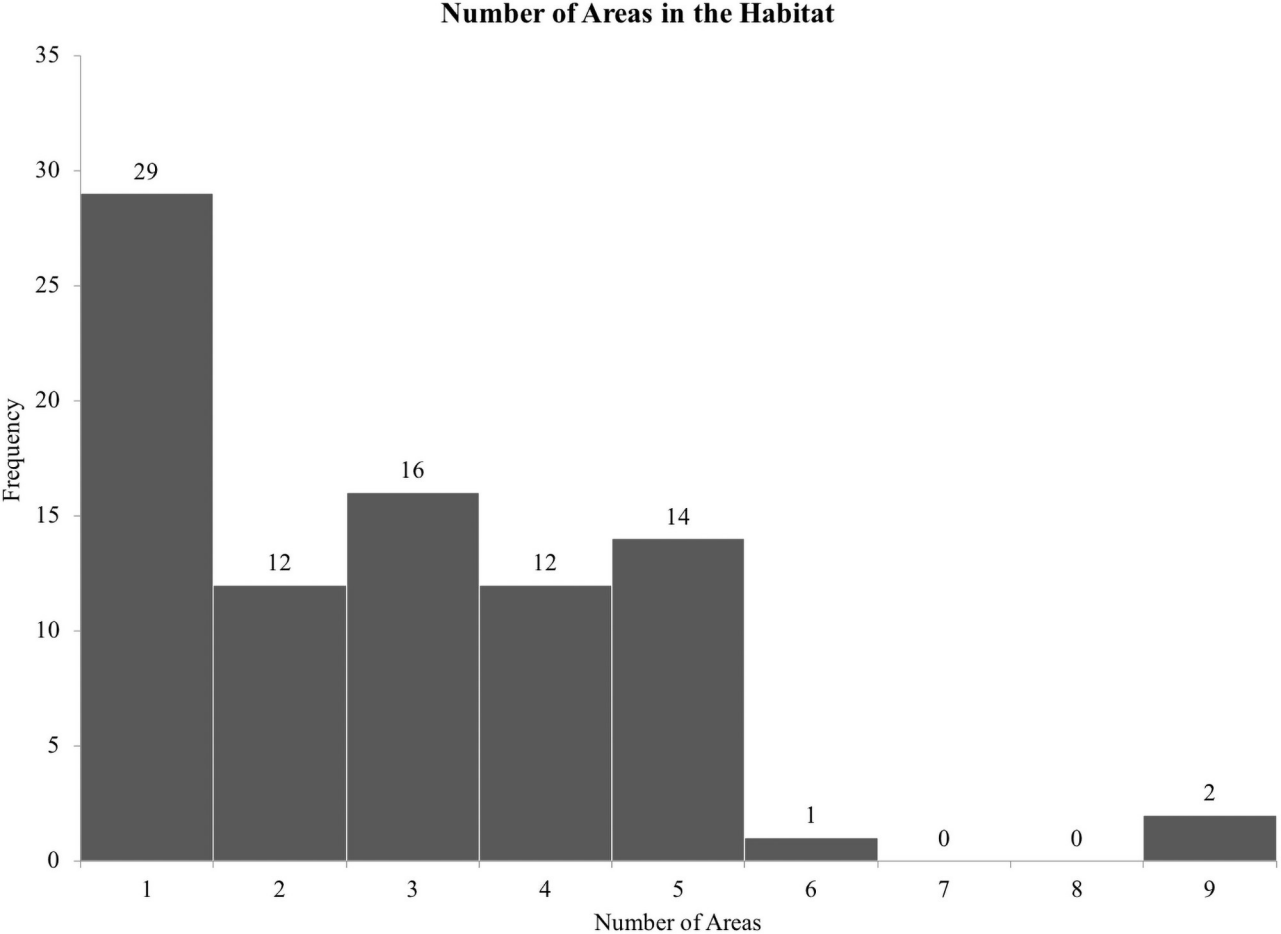
Number of areas in the habitat. The total number of areas the habitat could be separated into.

On average, dolphins had access to 2.61 megaliters (i.e., 689,489 gallons) of water at night and 2.54 megaliters (i.e., 670,997 gallons) of water during the day. There was a positive correlation between the maximum length of the habitat and daytime spatial experience (*r*(155) = 0.632, *p* < 0.01; Fig 2). Maximum depth was not related to daytime spatial experience (*r*(155) = -0.151, *p* = 0.06; Fig 3). The mean maximum habitat length was 41.28 m and the mean maximum habitat depth was 7.66 m with the majority of habitats less than 15 m deep and 70 m long (Fig 4). Twenty-four of the surveyed habitats were professionally managed zoo/aquarium habitats and 16 were professionally managed ocean habitats. Most facilities were designed so that areas of the habitat could be separated using a gating system. The majority of animals lived in habitats with five or fewer areas that could be separated by gates (Fig 5).

Fifty percent of dolphins were managed in the same group at all times. Thirty-three percent of dolphins were managed in small subgroups where individuals were rotated between subgroups. Seventeen percent of dolphins were managed in subgroups that were split and reunited. Thirty-seven percent of dolphins had visual and auditory access only (i.e., no physical contact) to other cetaceans in neighboring areas. Values for the direct and created independent variables relating to habitat characteristics are given in Table 5.

**Table 5:** Habitat characteristic variables.

Variable	Category	n	Mean	Median	SD	Min	Max	Inter-Quartile Range
Day Time Spatial Experience (Megaliter)		86	2.54	1.64	3.80	0.04	25.20	1.42
Night Time Spatial Experience (Megaliter)		86	2.61	1.63	3.76	0.38	25.20	1.62
24 Hour Spatial Experience (Megaliter)		86	2.55	1.60	3.76	0.38	25.20	1.47
Length (m)		86	41.28	37.00	20.19	9.14	120.00	16.25
Depth (m)		86	7.66	5.00	4.76	3.00	19.20	6.73
Habitat Type	Zoo/Aquarium Habitat	50	-	-	-	-	-	-
	Ocean Habitat	36	-	-	-	-	-	-
Number of Habitats		86	2.83	3.00	1.80	1.00	9.00	3.00
Social Management	Same Group	43	-	-	-	-	-	-
	Split/Reunited	28	-	-	-	-	-	-
	Rotated Subgroups	15	-	-	-	-	-	-
Neighboring Conspecifics	No Visual Access	54	-	-	-	-	-	-
	Visual/Auditory Access	32	-	-	-	-	-	-

## Discussion

Animal welfare science has evolved rapidly in recent decades and now employs a broad range of disciplines to evaluate welfare on both the individual and group level. In order to advance evidence-based management, it is necessary to quantify the features of current habitats, environmental enrichment programs, and training programs at a representative number of accredited facilities to better understand the variety of techniques used to manage bottlenose dolphins. Several factors including habitat characteristics, environmental enrichment programs, and training programs have been proposed as factors that may impact welfare outcomes. Here, we have detailed these factors across 40 habitats at 38 facilities.

One of the aims of the present study was to describe the scope of enrichment available at the facilities. While an individual must engage with the environmental enrichment for it to be effective, understanding the scope of options currently being made available to dolphins at accredited zoos and aquariums is still constructive. The vast majority of facilities utilized environmental enrichment that was included in the survey categories. Environmental enrichment for marine mammals can incorporate the addition of stimuli to meet species-appropriate needs including objects (e.g., balls and buoys; [24]), visual stimuli (e.g., television [47]), problems [18, 48], novel scents for appropriate species [49–51], and training sessions [52]. The effectiveness of the enrichment depends on a number of factors including presentation schedule and novelty [24, 25]. Prolonged exposure to enrichment can result in habituation and loss of effectiveness requiring constant evaluation and resetting of the enrichment [52, 53]. Enrichment presented on an intermittent basis is more likely to maintain its beneficial qualities and, therefore, it has been recommended that enrichment be presented on a variable basis [25]. In line with enrichment practices for other animals under professional care [54, 55], most respondents indicated that they scheduled their enrichment on a semi-random basis. Previous research has also suggested that novel objects are important additions to enrichment programs because they elicited increased attention [25]. The majority of the participating facilities regularly added new enrichment to their programs.

Managers consider a range of contributing factors when developing an enrichment program including but not limited to the age of the dolphins, the complexity of the physical and social environment, the physical health of the dolphin, and historical interactions with enrichment objects and activities. Similarly, the effectiveness of different types of enrichment is based on many factors including the animals social context and individual differences in preferences [22, 24]. One respondent indicated that the enrichment specified in the categories listed on the survey were not provided during the five-week data collection period. This facility may have provided enrichment that was not on the survey or leveraged naturally occurring types of enrichment to promote positive welfare and mental stimulation. For example, dolphins at one facility regularly engage in play with objects not included on the survey including vegetation (e.g., sea grass, seaweed, leaves, or seedpods), natural inanimate objects (e.g., wood/branches, sand, or rocks), non-fish species (e.g., crabs or lobster), and manipulation of dolphin-created bubbles that are in their environment [56–58]. These types of enrichment were not included as part of the survey because they are not always directly provided or controlled by animal care staff.

Established positive reinforcement training programs for bottlenose dolphins are an important part of maintaining optimal welfare [3, 29]. Modern training programs are founded in behavior analysis and operant conditioning techniques [59–61]. These programs emphasize the use of positive reinforcement and shaping through successive approximation to train and maintain behaviors [61]. Trained behaviors are used in several contexts such as educational presentations and interaction programs with the public, husbandry and medical settings, and research sessions. Dolphins have learned husbandry behaviors that allow them to participate in their own health care including, but not limited to, the collection of biological samples, dental work, ultrasounds, and completion of physical exams. Voluntary participation in their health care allows facilities to practice preventative medicine and aids in reducing stress when a dolphin requires treatment [10, 50]. For example, porpoises lifted from the water for health procedures had higher cortisol levels than those trained to voluntarily participate in the health procedure [29]. However, the elevation in cortisol is temporary and lifting animals may be occasionally required when an animal is ill and voluntary participation with training is no longer possible.

Training sessions have been associated with positive welfare indicators such as an increase in play behavior and behavioral diversity [3]. The predictability of the timing of training sessions may be an influential factor in behavior. The predictability of food related events has been studied in several species with inconsistent results. Studies based on behavioral measures found that predicable feeding schedules resulted in lower levels of stress, reduced anticipatory behaviors, and increased species-specific behaviors [62–66]. In contrast, predictable schedules have also been associated with increased rates of anticipatory behaviors that may lead to stereotypic behaviors [65–67]. Any changes in schedules should be undertaken with care because it has been demonstrated that moving from a predictable schedule to an unpredictable schedule can result in behavioral and physiological stress responses [63, 65].

Participants in the Cetacean Welfare Study included a large number of accredited facilities which were located over a broad geographical region. It is important to note that this study evaluated physical characteristics (i.e., length and depth) of the habitats that have been suggested to influence behavior [33, 34]. Within the sample population, 58.1% of dolphins lived in professionally managed zoo/aquarium habitats and 41.9% of dolphins lived in professionally managed ocean habitats connected to ocean water. In a 1997 survey of international cetacean facilities, 17.0% of habitats were classified as professionally managed ocean habitats [8].

Habitat use has been employed as a method for quantifying habitat suitability for a target species, including welfare outcomes for cetaceans under professional care [31, 32]. Habitats included in this study ranged from 9.14–120.00 m in maximum length and 3.00–19.20 m in maximum depth. Based on the areas the dolphins had access to during the day, the maximum length of the habitat appears to be more closely related to the total volume of water available when compared to the maximum depth. Dolphins had access to 0.07 ML (or 70 m^3^) more water volume on average during the night when compared to the day. While dolphins under professional care at one facility reduced their activity levels at night [68], the larger space available at night here suggested that any potential nighttime activities would not be hindered by space availability.

Dolphin habitats are designed, in part, to facilitate animal care professionals’ daily care and management of the animals. One way this goal is achieved is through the use of gates that can be opened and closed to divide the habitat. The participating facilities were able to separate their habitats into an average of 2.83 areas. These areas were used to manage access to the animals for the purposes of husbandry or veterinary care, to manage behavioral needs, and to maintain the desired social groups. The need for multiple areas may also stem from the sex, age, and breeding status of animals at a given facility. For example, it may be beneficial to maintain mother-calf dyads in a group separate from other adult dolphins during the early months of the calf’s life [69]. The social structure of some wild dolphins is based on a dominance hierarchy that influences individual and group dynamics with changes taking place during reproductive seasons [70, 71]. These evolutionary systems need to be taken into consideration when managing cetaceans under professional care because they can greatly influence the mental and physical welfare of the animals [16]. Welfare research can help to investigate and balance those overlapping factors to provide healthy lives for individuals where food acquisition is no longer requiring the majority of the animal’s time [4, 72, 73].

The results of this survey provided an overview of habitat features common to cetacean habitats in accredited facilities, as well as an outline of the general training and environmental enrichment programs at these facilities. The surveyed facilities provided a wide range of environmental enrichment which included both simple and complex activities that occurred above and under water. The majority of facilities provided the environmental enrichment on a semi-random schedule and regularly add new enrichment to their programs. All of the participating zoos and aquariums had training programs that included multiple training sessions every day on both predictable and semi-predictable schedules. The vast majority of dolphins participated in interaction programs with guests, public presentations, or both types of public engagement. The dolphins at surveyed facilities lived in both professionally managed zoo/aquarium habitats and professionally managed ocean habitats of varying size with a variety of social environments. Overall, the survey results revealed that accredited zoos and aquariums included habitats with a variety of characteristics and engaged in many common environmental enrichment and training practices.

The information gained from the management survey was also used in other research published from the Cetacean Welfare Study data set. The direct and synthesized metrics described here were leveraged for use in the following studies included within this collection [43–46, see [74] for a summary of these findings]. These works investigated important connections between potential indicators of welfare and habitat characteristics, demographic variables, environmental enrichment, and training practices. They also illustrated a trend in the management of cetaceans toward an increased understanding of species-appropriate habitats and the overlap necessary to blend physiological and psychological needs with human-made management systems. The information from this study can help facilities and personnel manage dolphins complex physical and mental needs through the application of basic scientific principles to evaluate current practices in order to continually improve the welfare of cetaceans.

## Supporting information

S1 Data (XLSX)

S1 File Cetacean welfare animal management survey. (DOCX)

S1 Fig (TIFF)
