# Supplementary material for: Environmental enrichment, training, and habitat characteristics of common bottlenose dolphins (Tursiops truncatus) and Indo-Pacific bottlenose dolphins (Tursiops aduncus)
Source: PLoS One. 2021 Aug 30;16(8):e0253688. doi: 10.1371/journal.pone.0253688 (PMC8404999; doi:10.1371/journal.pone.0253688)
Supplement: S1 File — (DOCX) [file pone.0253688.s002.docx]

**Cetacean Welfare Animal Management Survey**

**Habitat:**

1. Which type of habitat are the dolphins wearing the MTags housed in?
   1. Indoor habitat - artificial enclosure protected from the elements
   2. Outdoor habitat - artificial enclosure with exposure to weather patterns
   3. Closed ocean pen - natural ocean pens, dolphins never have access to the open ocean
   4. Semi-open ocean pen - natural ocean pens, dolphins may have access to the open ocean through gates (in or out of session)
2. Describe the areas of the habitat that the MTag dolphins have access to.
   1. Area 1
      1. Volume in Liters
      2. Maximum Length in Meters
      3. Maximum Width in Meters
      4. Maximum Depth in Meters
   2. Question repeated for additional areas

**Dolphin Information:**

1. Which best describes the typical schedule for social management?
   1. The animal remains in the same group at all times.
   2. The animal’s group is split into specific or rotating subgroups during the day and reunited at night.
   3. The animal’s subgroup is regularly rotated within a larger group (for example, a group of 10 animals managed in groups of 2-4 and individuals in the subgroup regularly rotate but the group of 10 is not united).
2. Is the dolphin housed adjacent to other dolphins that they are not allowed physical access to?
   1. Yes
   2. No
3. On average, how many hours during the day (open to the public) do they have access to the areas listed below?

| **Area/s** | **Number of hours** |
| --- | --- |
| Area 1 only |  |
| Area 2 only |  |
| Area 3 only |  |
| Area 1 and 2 |  |
| Area 1 and 3 |  |
| Area 2 and 3 |  |
| Area 1, 2, and 3 |  |
| Etc… |  |

1. On average, how many nights (closed to the public) a week do they have access to the areas listed below?

| **Area/s** | **Number of hours** |
| --- | --- |
| Area 1 only |  |
| Area 2 only |  |
| Area 3 only |  |
| Area 1 and 2 |  |
| Area 1 and 3 |  |
| Area 2 and 3 |  |
| Area 1, 2, and 3 |  |
| Etc… |  |

**Training Program:**

1. For structured public in-water programs (not free swims), what is the **maximum and average** number of guests allowed in the water?
   1. Maximum
   2. Average
   3. Not applicable
2. For public in-water free swim programs, what is the **maximum and average** number of guests allowed in the water?
   1. Maximum
   2. Average
   3. Not applicable
3. Which best describes how your facility schedules training sessions?
   1. Predictable – consistent daily session times
   2. Semi-predictable – intentionally varied session times
   3. Unpredictable – randomly scheduled using computerized randomization (e.g., a random number generator)

**Enrichment Program:**

1. Does your team set goals by identifying behaviors you want to encourage or discourage?
   1. Yes
      1. How often does your team set these goals?
         1. Daily
         2. Weekly
         3. Monthly
         4. Twice a year
         5. Yearly
   2. No
2. Does your team create enrichment plans to achieve the desired behavioral goals?
   1. Yes
      1. How often does your team create these goals?
         1. Daily
         2. Weekly
         3. Monthly
         4. Twice a year
         5. Yearly
      2. Which schedule best describes your enrichment plans?
         1. Predictable – Objects provided at the same time every day
         2. Semi-random – The items are purposely provided at different times.
         3. Random – The times objects are provided is scheduled randomly by a computer program.
   2. No
3. Does your team provide enrichment objects at night?
   1. Yes
      1. How many nights per week is the enrichment provided?
         1. 1
         2. 2
         3. 3
         4. 4
         5. 5
         6. 6
         7. 7
   2. No
4. Does your team record when enrichment is presented, and which objects are provided?
   1. Only when it is presented
   2. Only which objects are provided
   3. Both when and which objects
   4. Neither
5. Does your team evaluate the effectiveness of the enrichment?
   1. Yes
      1. How often is the enrichment evaluated?
         1. Once a week
         2. Once a month
         3. Twice a Year
         4. Yearly
      2. How often does your team adjust the enrichment plan based on the evaluation?
         1. Once a week
         2. Once a month
         3. Twice a Year
         4. Yearly
   2. No
6. Indicate the number of days in a typical 30 day month that the dolphins receive each type of enrichment.

| **Enrichment** | **Number of days** | **Average duration the object/s are given** |
| --- | --- | --- |
| Above water and scuba play |  |  |
| Balls and buoys |  |  |
| Boomer and beach balls |  |  |
| Bubble machines |  |  |
| Changing conspecifics |  |  |
| Dead fish |  |  |
| Feeder balls and spools |  |  |
| Foam rollers, bats, and sticks |  |  |
| Hula hoops |  |  |
| Ice and gelatin |  |  |
| Kayaking and Zorb balls |  |  |
| Legos and dive bricks |  |  |
| Live fish |  |  |
| Mats, sleds, and ice bergs |  |  |
| Mirror, television, and movies |  |  |
| Noodles |  |  |
| Puzzle feeders |  |  |
| Rub ropes and seaweed boas |  |  |
| Tubs |  |  |
| Underwater music or sounds |  |  |
| Underwater window play |  |  |
| Water spray and brush boards |  |  |
