# Supplementary figures and images for: Environmental enrichment, training, and habitat characteristics of common bottlenose dolphins (Tursiops truncatus) and Indo-Pacific bottlenose dolphins (Tursiops aduncus)
Source: PLoS One. 2021 Aug 30;16(8):e0253688. doi: 10.1371/journal.pone.0253688 (PMC8404999; doi:10.1371/journal.pone.0253688)

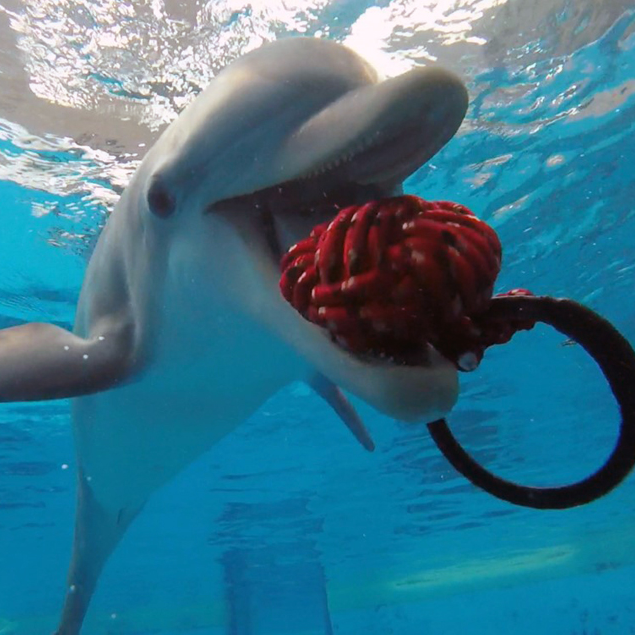

Supplement: S1 Fig — (TIFF) [file pone.0253688.s003.tiff]
